# Characterization of an H3N2 triple reassortant influenza virus with a mutation at the receptor binding domain (D190A) that occurred upon virus transmission from turkeys to pigs

**DOI:** 10.1186/1743-422X-7-258

**Published:** 2010-09-30

**Authors:** Hadi M Yassine, Mahesh Khatri, Chang W Lee, Yehia M Saif

**Affiliations:** 1 Food Animal Health Research Program, Ohio Agricultural Research and Development Center, The Ohio State University, 1680 Madison Ave, Wooster, OH 44691, USA; 2 Vaccine Research Center, National Institute of Allergy and Infectious Diseases, National Institutes of Health, 40 Convent Drive MSC 3005, Bethesda, MD 20892, USA

## Abstract

The hemagglutinin (HA) protein of influenza virus mediates essential viral functions including the binding to host receptor and virus entry. It also has the antigenic sites required for virus neutralization by host antibodies. Here, we characterized an H3N2 triple reassortant (TR) influenza virus (A/turkey/Ohio/313053/04) with a mutation at the receptor binding domain (Asp190Ala) that occurred upon virus transmission from turkeys to pigs in an experimental infection study. The mutant virus replicated less efficiently than the parental virus in human, pig and turkey primary tracheal/bronchial epithelial cells, with more than 3-log_10 _difference in virus titer at 72 hours post infection. In addition, the mutant virus demonstrated lower binding efficiency to plasma membrane preparations from all three cell types compared to the parental virus. Antisera raised against the parental virus reacted equally to both homologous and heterlogous viruses, however, antisera raised against the mutant virus showed 4-8 folds lower reactivity to the parental virus.

## Introduction

Influenza A viruses infect a wide range of animal species including mammals and birds [1]. All subtypes have been isolated from avian species, however, few subtypes have circulated and caused disease in mammals [2]. Generally speaking, avian viruses preferentially bind to N-acetylneuraminic acid-α2,3-galactose form of sialic acid (α2,3-S.A.) receptors while human viruses preferentially bind to α2,6-S.A. receptors [3].

The HA is a major surface glycoprotein on influenza virus envelope and is essential for binding to host receptors and virus entry [4]. In addition, it embraces the major immunogenic sites required for virus neutralization by host antibodies [5]. Previous studies have identified key residues at the receptor binding domain (RBD) of the HA molecule that are critical in determining host range specificity of influenza viruses. In H2 and H3 subtypes, Gln226Leu and Gly228Ser mutations accounted for shifting from avian to human receptor binding specificity [6,7]. In H1 subtypes, Glu190Asp and Gly225Glu mutations appear critical for adaptation of avian viruses to humans [8]. Neither of the mutations observed in H1 or H3 viruses, that caused a shift from avian to human receptor binding specificity, correlated with the shift in binding specificity of H5 viruses [9].

In this study, we characterized an H3N2 triple reassortant (TR) influenza virus with a mutation at the RBD (Asp190Ala) that occurred upon virus transmission from turkeys to pigs in an experimental infection study [10]. H3N2 TR viruses, which are characterized by having genes from human (HA, NA, and PB1), swine (NP, M, and NS) and avian (PB2, PA) lineage viruses, emerged in pigs in 1998 and then in turkeys in 2003 [11]. The HA of H3N2 TR viruses is originally of human lineage viruses [12], and swine isolates of this subtype retain Asp at residue 190 of the RBD. Similarly, turkey isolates express Asp at the corresponding position, except for two isolates from Minnesota that expressed Val (NCBI gene bank accession number: ACF25543) or Ala (NCBI gene bank accession number: ACD35865) at the corresponding position.

In general, avian viruses express Glu (specific for α2,3-S.A. receptors) and human viruses expresses Asp (specific for α2,6 S.A. receptors) at position 190 of the RBD [8,13]. Ala is rarely expressed at this position and characterization of such mutation is essential for its possible effect on antigenicity, receptor binding specificity, and interspecies transmission of H3 subtype influenza viruses [14-17].

## Materials and methods

### Generation of mutant viruses

The H3N2 TR virus used in this study, A/turkey/Ohio/313053/04 (TK04), was previously isolated at our laboratory [11] and has been propagated two times in 10-day-old embryonated chicken eggs (ECE).

Utilizing the 12-plasmid reverse genetics system, we rescued the TK04 virus as previously described [18,19]. Briefly, the HA, NP, NA, M, and NS genes were amplified with one-step RT-PCR kit (Qiagen, Valencia, CA), while the polymerase genes (PB1, PB2, and PA) were amplified with two-steps RT-PCR, using SuperscriptIII and Elongase Enzyme, respectively (Invitrogen, San Diego, CA). PCR products were purified and digested with *Bsm*BI restriction enzyme and cloned into pHH21 vector between promoter and terminator sequences of RNA polymerase I. Eight plasmids harboring the eight gene-segments were transfected along with four expression plasmids (pCAGGS-WSN-NP, pcDNA774-PB1, pcDNA762-PB2, and pcDNA787-PA, kindly provided by Dr. Y. Kawaoka, University of Wisconsin, Madison, WI) into 293T cells with the help of Lipofectamine-2000 reagent (Invitrogen, San Diego, CA). Supernatant from transfected cells was collected at 36 hours post transfection (hpi) and was subsequently inoculated into 10-day-old ECE for virus isolation. Single amino acid change at residue 190 of the RBD (Asp to Ala) was generated using QuikChange^® ^Site-Directed Mutagenesis kit **(**Stratagene, La Jolla, CA) based on manufacture protocol. In addition, we generated a virus with a mutation at residue 627 of PB2 gene (Glu627Lys) that has been shown to affect replication and transmission of influenza viruses in different species [20].

### Assessment of virus replication in human, pig, and turkey tracheal/bronchial epithelial cells

Primary human tracheal/bronchial epithelial cells (HAEC) were purchased from Cell Application (Cell Application, San Diego, CA) and were maintained in tracheal/bronchial epithelial cells growth medium purchased from the same company (catalogue no. 511-500).

Primary pig and turkey tracheal/bronchial epithelial cells (PEC and TEC, respectively) were generated based on previously published protocols with slight modifications [21-23]. Briefly, distal-tracheal/proximal-primary bronchial airway tissues were collected from 5-weeks old healthy pig or 1-day old specific pathogen free (SPF) turkey. Tissues were cut into small fragments (~1 cm long) and were treated with pronase enzyme (1.4 mg/ml, Boehringer Mannheim, Indianapolis, IN) for 24-48 hours at 4°C. Pronase activity was stopped by adding 10% FBS in DMEM medium, cells were washed with PBS and then suspended in serum free mammary epithelial growth media supplemented with bovine pituitary extract, human epidermal growth factor, insulin and hydrocortisone (MEGM, Lonza, Walkersville, MD). To remove contaminating fibroblasts, cells were incubated for 2-4 hours at 37°C and 5% CO_2 _and non-adherent epithelial cells were collected and seeded into new culture flask for further growth. Cells were passaged up to five times prior to use in experiments.

For the kinetic study, 70-80% confluent cells seeded in 6-well plate were infected with either virus at 0.01 TCID_50_. Serum free DMEM media served as negative control. Plates were rocked every 15 minutes and inoculum was removed after 45 minutes followed by adding DMEM media supplemented with 1 μg/ml TPCK-treated trypsin on top of the cells. Supernatant from inoculated cells was collected at 24, 48, and 72 hpi and titrated in Madin-Darby canine kidney (MDCK) cells based on previously published protocol [24]. Data were analyzed using graphPad prism software (GraphPad Software, Inc., La Jolla, CA, USA) by applying paired t-test with 95% confidence interval.

### Assessment of cross reactivity between parental and mutant viruses

The cross hemagglutinin inhibition (HI) test was employed to evaluate the cross reactivity between parental (190Asp) and HA-mutant (190Ala) TK04 viruses. Additionally, cross reactivity was evaluated between TK04 parental and mutant viruses, and other H3N2 TR viruses isolated from turkeys in the United States (U.S.). This includes: A/turkey/North Carolina/03, A/turkey/Illinois/04, A/turkey/Minnesota/05, and A/turkey/North Carolina/05.

Antisera against TK04 viruses were produced by vaccinating two 2-week-old chickens with an inactivated virus vaccine (oil emulsion, 10^6 ^TCID50/ml) for three times in 2-weeks interval. HI test was carried out as previously described [25]. Briefly, titers were determined by using two-fold serially diluted serum (25 μl), 4 HA units (25 μl) of homologous or heterologous antigen, and a 1% (50 μl) suspension of turkey erythrocyte per test well.

The antigenic relatedness between the different viruses was expressed as R-value based on the Archetti and Horsfall formula [12,26]. The R-value is equivalent to the square root of r1 × r2, where r1 is the ratio of heterologous titer obtained with virus 2 to homologous titer obtained with virus 1; r2 is the ratio of the heterologous titer obtained with virus 1 to homologous titer obtained with virus 2.

### Plasma membrane binding assay

Plasma membranes were prepared from HAEC, PEC, and TEC based on formerly published protocol [27-29]. Solid phase binding assay [30] was carried out as follows: plasma membrane preparations (PMP) were coated into 96-well plate (Costar, Lowell, MA) at concentration of 25 μg/ml overnight at 4°C. Plates were rinsed with PBS and then blocked with 0.2% BSA in PBS for 2 hours at 37°C. Two-fold serially diluted virus (50 μl; 64-4 HA) in reaction buffer (0.02% BSA in PBS) were added to wells and incubated at 4°C for one hour. Wells not coated with plasma membranes but blocked and treated with virus as indicated above were used as negative controls. Plates were then washed four times with ice-cold washing buffer (0.2XPBS containing 0.05% tween-80), followed by addition of 50 μl/well of peroxidase-labeled fetuin for 1 hour at 4°C. After four washes as indicated above, color was developed by adding 100 μl SureBlue TM-TMB substrate (KPL, Gaithersburg, MD) for 10 min at 37°C. The reaction was stopped with 100 μl 2N H_2_SO_4 _and OD_450 _nm measurement was obtained. Dose-response curves were generated by plotting OD_450 _nm values on *y*-axis and virus concentration (in HA units) on *x*-axis. To inhibit neuraminidase activity, all experiments were performed in the presence of Zanamivir hydrate (Moravek, CA, USA) at a final concentration of 0.25 μm. Recorded results are the average of three independent experiments.

## Results and discussion

In 1998, a new subtype of influenza A viruses, H3N2 TR, emerged in pig population in the U.S. and transmitted to other species including humans, turkeys, minks and waterfowls [11,31-33]. In a previous study performed by our group, we evaluated the replication and transmission of H3N2 TR viruses between avian and mammalian species. Viruses that shared more than 99% of their genome sequences behaved differently in terms of transmission between swine and turkeys [10]. Only one virus (A/turkey/Ohio/313053/04) transmitted efficiently both ways between swine and turkeys. Another virus (A/turkey/North Carolina/03) transmitted one way from pigs to turkeys but not vice verse. Neither of other two viruses (A/turkey/Illinois/04 and A/swine/North Carolina/03) transmitted either way between the two species. One of these viruses, TK04, which transmitted both ways between pigs and turkeys, expressed changes at or close to the RBD of the HA molecule upon transmission between the two species [10].

**Figure 1:**
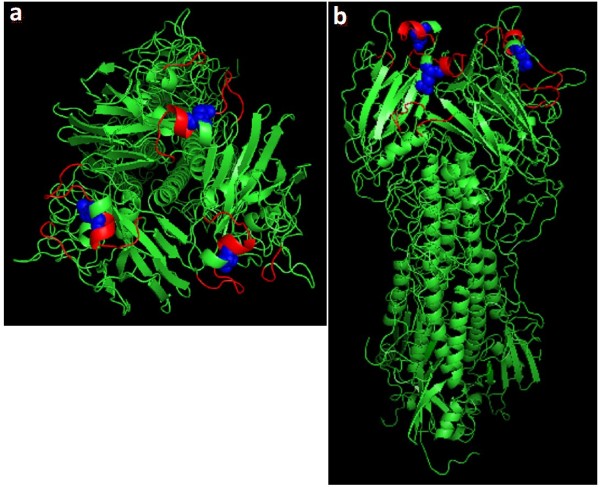
**HA structure with Asp to Ala mutation at residue 190 of the RBD**. The 3D structure of the HA molecule was downloaded from Protein Data Bank webpage (http://www.pdb.org; 1HGG-A/Aichi/2/68 (H3)) and modified using the PYMOL Molecular Graphics System (DeLano Scientific, San Carlos, CA). a: top view of the HA molecule; b: side view of the HA molecule. Red: RBD. Blue balls: Residue 190 of the RBD.

One change, Asp to Ala, occurred at residue 190 of the RBD (Figure 1) upon virus transmission from turkeys to pigs. Several studies have shown the importance of this residue in determining the receptor binding specificity and host range of influenza A viruses. Most of these studies were performed with the 1918 pandemic-H1N1 virus or highly pathogenic H5-subtype viruses [9,14,16], and work has not been done to characterize this residue in the swine lineage H3-subtype viruses. Hence, we initiated this study to evaluate the effect of Asp190Ala mutation on H3N2 TR virus behavior *in vitro *utilizing reverse genetics created viruses.

First, we evaluated the replication of TK04 parental and HA-mutant viruses (hereafter referred as 190Asp and 190Ala, respectively) in human, pig and turkey primary tracheal/bronchial epithelial cells. Virus with a mutation at residue 627 of the PB2 gene (Glu627Lys) was used as control, where such mutation has been shown to affect replication and host range specificity of influenza viruses.

**Figure 2:**
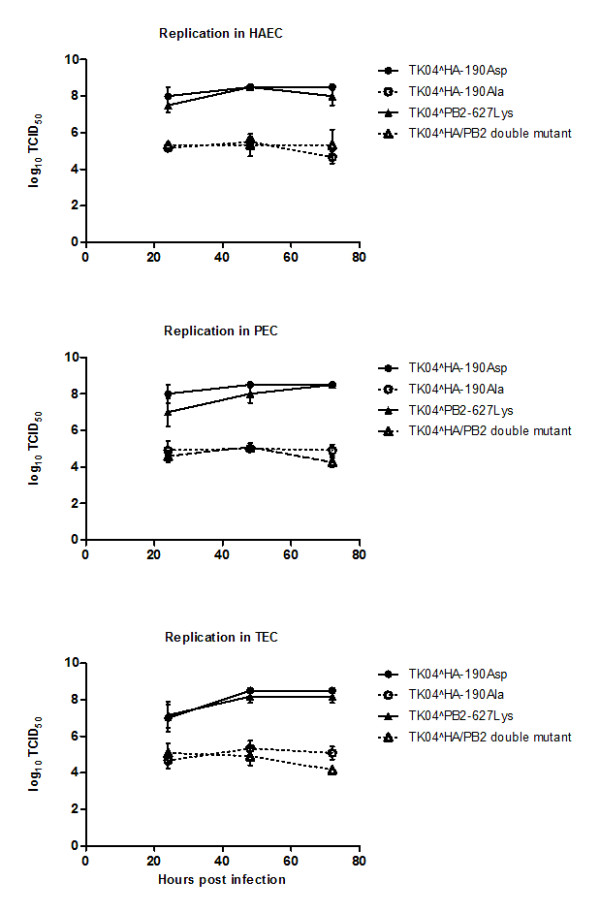
**Replication of parental and mutant TK04 viruses in human, pig and turkey primary tracheal/bronchial epithelial cells**. Parental virus has Asp at residue 190 of the RBD, while the mutant virus has Ala at the corresponding position. A strain with Glu627Lys mutation in the PB2 gene was included in the kinetic study to serve as control, since such mutation was shown to affect host range specificity of influenza A viruses. Parental TK04-190Asp replicated more efficiently than the mutant TK04-190Ala in three cell types (P-values <0.0091, <0.0021, and <0.0119 for HAEC, PEC and TEC respectively). Mutation in the PB2 gene did not affect virus replication.

The 190Asp virus replicated more efficiently than 190Ala virus in the three cell types of mammalian and avian origin (P-values <0.0091, <0.0021, and <0.0119 for HAEC, PEC and TEC respectively). Evident variation in virus titer was manifested since 24 hpi, with more than 3-log_10 _difference in virus titer between 190Asp and 190Ala viruses recorded at 72 hpi (Figure 2). Interestingly, Glu627Lys mutation in the PB2 gene did not affect virus replication in all three cell types (Figure 2), supporting a recent finding which indicated that Glu627Lys substitution in PB2 gene does not increase virulence nor growth rate of pandemic-H1N1 (2009) virus in mice and cell culture [34]. It is worth noting that the PB2 gene of H3N2 TR and pandemic-H1N1 viruses is originally of avian lineage viruses and it maintains avian like residue (Glu) at the corresponding position.

**Figure 3:**
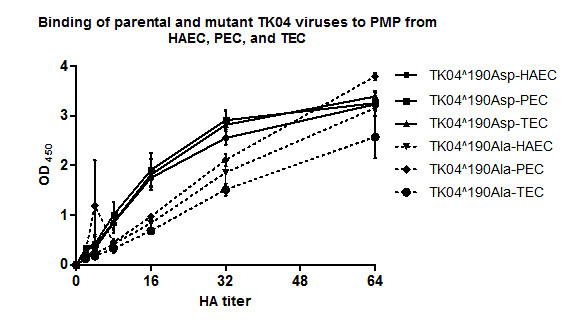
**Binding of parental TK04-190Asp and mutant TK04-190Ala viruses to plasma membrane preparations (PMP) from human, pig and turkey primary tracheal/bronchial epithelial cells**. Both viruses bound with similar efficiency to PMP from HAEC and PEC but not TEC (P-value < 0.02) at high virus titer (64 HA). Nonetheless, 190Ala virus showed decreased binding efficiency (P-value <0.04 and <0.019 for HAEC and PEC respectively) to all PMP at lower titers.

We then assessed the effect of Asp190Ala mutation on binding efficiency of the TK04 virus to PMP from primary tracheal cells of human, pig and turkey origin (Figure 3). Both viruses (190Asp and 190Ala) bound with similar efficiency to PMP from HAEC and PEC but not TEC (P-value < 0.02) at high virus titer (64 HA). Nonetheless, 190Ala virus showed decreased binding efficiency (P-value <0.04 and <0.019 for HAEC and PEC respectively) to all PMP at lower titers, with two-fold difference recorded at 16 HA compared to the parental-190Asp virus (Figure 3).

Next, we evaluated the effect of Asp190Ala mutation on antigenicity of H3N2 TR virus using the conventional cross-HI test (Table 1). Anti-190Asp antisera reacted equally to both 190Asp and 190Ala viruses. On the other hand, anti-190Ala antisera exhibited 4-8 folds less reactivity to the heterologous parental-190Asp virus.

**Table 1:** Cross reactivity between TK04 parental (190Asp) and mutant (190Ala) viruses as well as other H3N2 TR viruses of turkey origin based on HI-test.

Serum Virus	Anti-TK04(190Asp)	Anti-TK04(190Ala)	Anti-NC03	Anti-IL04	Anti-MN05	Anti-NC05
**TK04(190Asp)**	2048/1024*	256/128*	128	64	1024	512
**TK04(190Ala)**	2048/1024*	1024/1024*	64	64	2048	512
**NC03**	512	256	*64*			
**IL04**	2048	256		*128*		
**MN05**	4096	2048			*4096*	
**NC05**	2048	512				*1028*

* Antisera against each virus were produced by vaccinating two 2-week-old chickens with an inactivated virus for three times in two weeks interval.

To further evaluate the above results, we included a wider range of turkey H3N2 TR viruses in the cross reactivity test. Again, anti-190Asp antisera reacted better against most turkey viruses compared to anti-190Ala antisera (Table 1). For example, Anti-190Asp showed similar reactivity to IL04 and homologous viruses, where both viruses share more than 98% of the HA protein sequences [12], including residue 190 of the RBD. However, Anti-190Ala exhibited four-fold lower reactivity to IL04 compared to the homologous virus. On the other hand, both antisera exhibited two-fold increase in reactivity to a 2005 strain from Minnesota (MN05) compared to homologous viruses. Interestingly, MN05 virus has been published to have similar mutation at residue 190 of the RBD (NCBI gene bank accession number: ACD35865), and thus, supporting the effect of such mutation on the antigenicity of H3N2 TR viruses.

To have a better interpretation of the above observations, we translated the HI-cross reactivity results to "percent antigenic relatedness (R)" between the different viruses using the Archetti and Horsfall formula [26]. The parental-190Asp and mutant-190Ala viruses showed 50% antigenic similarity (Table 2). While the parental-190Asp exhibited around 71% similarity to all H3N2 TR viruses, the R-values decreased to 50% or less between the mutant-190Ala and other H3N2 viruses (Table 2). Expectedly, the MN05 strain displayed 100% antigenic similarity to 190Ala virus, as a result of expression of the same amino acid (Ala) at position 190 of the HA-RBD.

**Table 2:** Cross-HI results expressed as percent antigenic relatedness (R*)

	TK04(190Asp)	TK04(190Ala)
**TK04(190Asp)**	*100*	50
**TK04(190Ala)**	50	*100*
**NC03**	71	50
**IL04**	71	35
**MN05**	71	100
**NC05**	71	50

R-values were calculated based on Archetti an Horsfall formula. R = square root (r1 × r2), where r1 is the ratio of heterologous titer obtained with virus 2 to homologous titer obtained with virus 1; r2 is the ratio of the heterologous titer obtained with virus 1 to homologous titer obtained with virus 2.

Although antibodies to the HA-antigenic sites have been shown to affect receptor binding specificity and neutralization sensitivity, mutations solely to the RBD have not been shown to alter immunogenicity [16]. In this paper, we report on naturally occurring mutation at the RBD of the HA molecule that affect antigenicity, binding efficiency, and replication competence of H3-subtype viruses.

Glu (specific for α2,3-S.A. receptors) is typically expressed in avian viruses at residue 190 of the HA molecule, while human viruses express Asp (specific for α2,6-S.A. receptors) at the corresponding position. Both amino acids are negatively charged, while Ala is a neutral amino acid. We assume that Ala at the corresponding position (Figure 1) might not affect the configuration, but rather the charge at RBD, explaining in part the above observed results. Hence, viruses with Ala at residue 190 of the RBD can survive in nature although with less fitness compared to 190Asp expressing viruses.

In conclusion, the Asp190Ala mutation that occurred upon virus transmission from turkeys to pigs could have been a transient or rare occurring mutation that resulted in a less fitted virus, explaining the rareness of Ala at this position in swine and turkey H3N2 influenza isolates. More work is needed to evaluate the replication and antigenicity of 190Ala mutation *in vivo*. Additionally, it is of importance to see the effect of the above mutation on the receptor binding specificity of H3 subtype viruses for its potential effect on interspecies transmission of influenza viruses.

## Competing interests

The authors declare that they have no competing interests.

## Authors' contributions

YMS is the leader of the study group. HMY carried out the experiments and wrote the manuscript. MK generated the pig and turkey epithelial cells and helped in the infection studies. HMY, CWL, and YMS designed the experiments and analyzed the data. All authors read and approved the final manuscript.
